# Pivotal Cytoprotective Mediators and Promising Therapeutic Strategies for Endothelial Progenitor Cell-Based Cardiovascular Regeneration

**DOI:** 10.1155/2016/8340257

**Published:** 2016-12-20

**Authors:** Hyunyun Kim, Sujin Kim, Sang Hong Baek, Sang-Mo Kwon

**Affiliations:** ^1^Laboratory for Vascular Medicine and Stem Cell Biology, Convergence Stem Cell Research Center, Medical Research Institute, Pusan National University School of Medicine, Yangsan, Republic of Korea; ^2^Laboratory of Cardiovascular Regeneration, Division of Cardiovascular Medicine, Seoul St. Mary's Hospital, The Catholic University of Korea School of Medicine, Seoul, Republic of Korea

## Abstract

Cardiovascular diseases (CVDs), including atherosclerosis, stroke, and myocardial infarction, is a major cause of death worldwide. In aspects of cell therapy against CVD, it is generally accepted that endothelial progenitor cells (EPCs) are potent neovascular modulators in ischemic tissues. In response to ischemic injury signals, EPCs located in a bone marrow niche migrate to injury sites and form new vessels by secreting various vasculogenic factors including VEGF, SDF-1, and FGF, as well as by directly differentiating into endothelial cells. Nonetheless, in ischemic tissues, most of engrafted EPCs do not survive under harsh ischemic conditions and nutrient depletion. Therefore, an understanding of diverse EPC-related cytoprotective mediators underlying EPC homeostasis in ischemic tissues may help to overcome current obstacles for EPC-mediated cell therapy for CVDs. Additionally, to enhance EPC's functional capacity at ischemic sites, multiple strategies for cell survival should be considered, that is, preconditioning of EPCs with function-targeting drugs including natural compounds and hormones, virus mediated genetic modification, combined therapy with other stem/progenitor cells, and conglomeration with biomaterials. In this review, we discuss multiple cytoprotective mediators of EPC-based cardiovascular repair and propose promising therapeutic strategies for the treatment of CVDs.

## 1. Introduction

Excessive nutrient intake from food affects public health [1, 2]. In particular, immoderate intake of salt [3], fat [4], and sugars [5] is closely related to cardiovascular diseases (CVDs). These CVD-inducing factors are present in blood and circulate with blood. High concentrations of sodium, lipids, and glucose require additional blood to sustain blood homeostasis [6]. To pump blood as a routine task, the heart requires enhanced contractile force. This process strains the heart and causes cardiac diseases including angina [7], cardiac infarction [8], and arrhythmia [9] as well as high blood pressure [10] and onset of damage to vessels. In addition, excessive nutrient causes pathogenesis of CVDs. For instance, over-intake lipids are deposited in the arterial blood vessel and narrow the vessel diameter. Endothelial inflammatory mechanism is activated, sequentially triggering migration of inflammatory cells toward the lipid-accumulated site of blood vessel. These cells ingest lipid and transform themselves into foam cells, a pathologic elements of atheroma [11], which are conjugated with smooth muscle cells (SMCs) and generate fibrous extracellular matrix in the lesions. Cap-like structure of mixture is weakened by the proteolytic enzyme from inflammatory cells and easy to rupture [12]. Although blood vessels maintain their physical condition, the loss and insufficient durability of blood vessels cause CVDs, including atherosclerosis [13], stroke, and ischemia [14]. To identify the best therapeutic approach to CVDs, traditional studies have been focused on pharmacotherapy of CVDs, with an obvious limitation incomplete functional recovery from a CVD as well as side effects including diarrhea, rash, or itching. Recently, advances in stem cell biology, directly targeting potent cytoprotective mediators in injured tissues via an* in situ* transplant of stem and progenitor cells, have highlighted the strong potential of stem cell-based therapy against ischemic CVDs.

In 1997, Asahara et al. discovered the presence of endothelial progenitor cells (EPCs) in human blood. EPCs reside in a bone marrow (BM) niche and interact with neighboring cells or niche-forming cells. In response to ischemic signals, these progenitors are dramatically mobilized to blood vessels and are incorporated into injury sites [15]. EPCs engrafted in ischemic tissue then differentiate into their designated cell types: endothelial cells (ECs) or SMCs. Impaired vascular tissues are replaced with newly arriving and differentiated cells [16]. During the process of recovery from injury, pivotal cytoprotective mediators including well-known signaling pathways such as HIF-1*α*-dependent signals, Akt-dependent prosurvival signals, eNOS signaling, stromal-cell-derived factor 1*α*-dependent tissue incorporation signals, and several growth factors including vascular endothelial growth factor (VEGF) and fibroblast growth factor (FGF) have recently been reported in studies on EPC biology [17, 18].

In studies aimed at better clinical use of EPCs against ischemic CVDs, accumulating data have recently shown stronger therapeutic strategies involving targeting of these cytoprotective modulators, which may be linked to cell-cell interactions, cell-extracellular matrix (ECM) interactions, and upregulated proangiogenic cytokines and intracellular survival signals in ischemic tissues [19]. One of the most straightforward ideas for enhancing EPC function is the drug-based preconditioning strategy before the cell transplant into an ischemic site [20]. Compared to commercial drugs, natural compounds have some advantages, because they can be easily isolated from animals or plants and have less harmful side effects than artificial drugs. The other method is to enhance cell survival via genetic modification of the transplanted cells using an improved vector delivery system. Recently, retrovirus- [21], lentivirus- [22], and adeno-associated virus- (AAV-) mediated gene delivery systems [23] were shown to have distinct mechanisms, advantages, and flaws. A combinatorial cell therapy using two types of cells might be another powerful recovery enhancing strategy [24]. For example, a combination of EPCs and supporting cells may contribute to regeneration of not only vascular tissue but also other tissues including neurons, muscle, bone, and pancreas because restored blood vessels are capable of supplying sufficient blood with nutrients to a damaged tissue with effectively recruited hybridized partner cells including mesenchymal stem cells (MSCs) and *β*-cell islets [25]. Another emerging topic in stem cell therapy is artificial niche-mediated strategies involving transplanted cells including three-dimensional (3D) spheroid cells, artificial tissues constructed by 3D printing technologies, and biomaterial-based scaffold strategies, which may consist of living-organism-friendly niche components and protect EPCs from a harsh ischemic environment and nutrient depletion [26, 27].

In this review, we describe recent progress of EPC biology, discuss multiple topics on cytoprotective mediators of EPC-based cardiovascular repair, and offer promising therapeutic strategies for the treatment of CVDs.

## 2. EPC-Based Cardiovascular Repair

### 2.1. The Basics of EPC

In 1997, Asahara et al. first isolated EPCs from adult peripheral blood [28]. In addition to peripheral blood, EPCs can be isolated from various sources including BM [29], cord blood (CB) [30], fetal liver [31], and skeletal muscle [32]. These rare populations of cells can be mobilized into the circulation by diverse stimuli [33]. In response to growth factors and cytokines released during atherosclerosis [34], myocardial infarction [35], wound healing [36], limb ischemia [37], or tumor angiogenesis [38], EPCs are capable of homing to sites of a damaged endothelium or extravascular tissue and producing abundant angiogenic cytokines for promotion of* in situ* cell proliferation and vascular cell lineage differentiation; EPCs also directly differentiate into mature vascular endothelial cells. They are isolated as CD34^+^ cells from human peripheral blood and are cultured in a plate with a fibronectin-coated surface, forming endothelial-like cells [28]. After a few hours, early EPCs express typical EPC markers including CD34 (mucosialin) [39] and vascular endothelial growth factor receptor-2 (VEGFR-2) [40, 41]. Particularly, hematopoietic stem and progenitor cells coexpress a marker of immature human stem cells, CD133, also called the early hematopoietic-stem cell marker. In contrast to the progenitor marker CD34, mature endothelial cells or endothelial colony forming cells (ECFCs) do not express CD133 [42]. For this reason, a combination of these three markers of CD34^+^, CD133^+^, and VEGFR-2^+^ was recently reported as a promising EPC marker by some research groups [43, 44]. Nonetheless, identification of the unique surface marker of EPCs is still a controversial topic, which should be addressed in the near future [43, 45, 46].

The heart and circulatory system need a sufficient EPC number to keep the body healthy. Schmidt-Lucke et al. have studied the correlation between the number of circulating EPCs and future cardiovascular events in patients [47]. They tried to trace circulating EPCs with defined surface markers CD34 and KDR by flow cytometry; the 120 individuals were followed up for 10 months. Decreased numbers of EPCs were found to be associated with a higher incidence of CVDs. Jie et al. have reported a tight link between hemodialysis and circulating EPC numbers in children with chronic kidney disease [48] after studying CD34^+^KDR^+^ EPC numbers by flow cytometry. Children with chronic kidney disease [49] on hemodialysis showed 47% lower EPC levels as compared with the control. Compared to the hemodialysis chronic kidney disease group, however, children with predialysis chronic kidney disease showed no significant change. This result is in line with studies on adult patients [50]. Therefore, the uremic environment in patients with chronic kidney disease does not reduce EPC levels, but a reduced number of circulating EPCs can primarily contribute to CVD or coronary artery disease (CAD) [51]. Similarly, Eizawa et al. showed that patients with CAD have reduced number of circulating EPCs [52]. Using flow cytometry, those researchers demonstrated that circulating CD34^+^ cells are significantly downregulated in patients with CAD and the cell number is approximately 30% lower than that among age-matched control subjects.

### 2.2. Two Types of EPCs: Early EPCs and Late EPCs

Diverse research groups have isolated* ex vivo*-cultured EPCs from various tissues by different culture methods and characterized EPCs by means of distinct cell surface markers and endothelial-lineage-related functional assays. According to recent progress in EPC biology, EPCs are subdivided into two types: early EPCs and late EPCs. Early EPCs have spindle-shaped morphology with a short lifespan. These early EPCs strongly express CD45 [53], vascular endothelial growth factor receptor 1 (Flt1 or VEGFR1) [54], endothelial nitric oxide synthase (eNOS) [55], Von Willebrand factor (vWF) [56], and CD31 [57]. After 3 weeks, the expression of KDR and VE-cadherin disappears and cells die. On the other hand, late EPCs were obtained by prolonged incubation of peripheral mononuclear cells (MNCs) in the presence of VEGF [58]. These cells' morphology changes to cobblestone monolayer-like morphology of human umbilical vein endothelial cells (HUVECs) [59], but their proliferation potential is higher than that of HUVECs. Gene expression profiles of early EPCs and late EPCs also show difference. The expression levels of surface marker genes including CD14, Flt-1, and KDR [60] in early EPCs are inconsistent among reports by many groups [61] because early EPCs are a heterogeneous subpopulation. Nevertheless, gene expression profiles of late EPCs are quite similar to the profile of HUVECs. Compared to early EPCs, the KDR expression level is higher in late EPCs. Therefore, late EPCs show better capacity for formation of tubule-like structures and a stronger long-term survival potential in comparison with early EPCs. Early EPCs, however, can contribute to neovascularization by secreting greater amounts of angiogenic factors such as VEGF and interleukin 8 (IL-8) [62] than late EPCs do.

## 3. Understanding EPC-Mediated Vascular Repair

### 3.1. BM Niche as a Hometown of EPCs

Because EPCs and hematopoietic stem cells (HSCs) originate from derived from a hemangioblasts [63, 64], EPCs share diverse stem/progenitor specific markers with HSCs such as CD34, CD133, c-kit [65], and Sca-1 [66, 67]. Just as HSCs, EPCs mainly develop in BM [68] and are located in the osteoblastic niche, endosteal niche [69], and vascular niche [70]. Stem cells in the osteoblastic niche are recruited to the vascular niche via cytokines such as SDF-1. Thereafter, the migrating cells differentiate into committed progenitors as needed and set the stage for full reconstitution of BM [71]. Signaling pathways such as Ang-1/Tie2 [72], stem cell factor (SCF)/c-Kit [73], SDF-1/CXCR4 [74], and Jagged/Notch [75] of HSCs in BM niches play a critical role in a niche [76], whereas the origin of EPCs and their lineage determination in BM are not fully understood.

The undifferentiated progenitor cells including HSCs and EPCs also express c-Kit receptor, an immaturity marker. Heissig et al. recently reported the niche-modulation signals of stem/progenitor cells [77]. After myelosuppression by means of 5-fluorouracil, the expression level of matrix metalloproteinase-9 (MMP-9) is increased, and thereby MMP-9 promotes release of sKitL from the membrane form (mKitL). Furthermore, myelosuppression significantly induces stem cell mobilizing cytokines including SDF-1, G-CSF, and VEGF and stimulates a release of pro-MMP-9 and migration of human CD34^+^ stem/progenitor cells. Indeed, increased SDF-1 and VEGF levels in the plasma of wild-type (WT) mice enhance mobilization of circulating EPC, suggesting that recruitment and mobilization of BM-derived circulating EPCs require activation of MMP-9.

**Figure 1:**
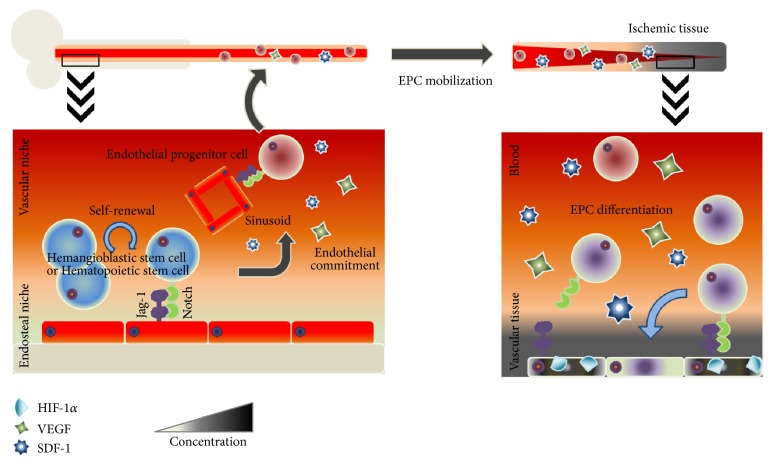
EPC homing from a BM niche to the circulation. HSCs and EPCs interact with stromal niche cells via Notch signals. HIF-1*α*, stabilized from ischemic signals, promotes EPC homing-related cytokines, including VEGF and SDF-1.

Another pivotal cytoprotective modulator in BM-EPC biology contributing to functional kinetics of the BM microenvironment is the Notch signaling pathway [78]. Although many research groups have studied Notch signaling pathways with respect to stem cell biology, relations between BM- and EPC-mediated vasculogenesis have not been fully elucidated [79]. Kwon et al. studied niche modulation of Jagged-1-mediated Notch signaling in a BM microenvironment [80]. Notch ligands (Jag-1 and Dll-1) are expressed by these niche-supporting cells [81]. Compared to WT and Dll-1^−/−^ mouse cells, Jag-1^−/−^ cells form fewer EPC colonies. This result is similar to the finding that *γ*-secretase II, a blocker of cleavage of the intracellular domain in Notch receptor, mediates inhibition of Notch signaling in BM-KSL cells (Kit^+^Sca-1^+^Lin^−^). To test the contribution of Notch signals to recovery from ischemia, they generated a hindlimb ischemia model and transplanted EPCs from BM of each KO mouse. Unlike BM EPCs from WT and Dll-1^−/−^ mice, BM EPCs derived from Jag-1^−/−^ mice fail to augment blood perfusion, suggesting that EPCs are affected by the niche microenvironment of BM, and EPCs preconditioned by a specific Jag-1-dependent signaling pathway may be useful for therapeutic neovascularization [81] (Figure 1).

The Lnk adaptor protein with a Src homology 2 domain has been studied in B cells and HSCs [82, 83]. This gene is highly expressed in immature cells and act as a negative regulator of the SCF/c-Kit signaling. On the basis of the findings of this study, Kwon et al. also investigated the cytoprotective role of Lnk in EPC biology [84]. The* lnk* expression levels were found to be higher in BM hematopoietic and endothelial progenitors than among other differentiated BM cells. Specific deletion of the* lnk* gene results in an increase in immature subpopulation of KSL (Kit^+^/Sca-1^+^/Lin^−^) cells and significantly upregulates endothelial markers including Flk-1, VE-cadherin, CD31, and CXCR4 in lnk^−/−^ mice but not WT mice. Blood flow in the hindlimb ischemic region containing transplanted EPCs is significantly enhanced in lnk^−/−^ mice. Moreover, EPC survival-related genes, Akt and eNOS, are also upregulated in lnk^−/−^ mice, providing strong evidence that the Lnk adaptor protein is a downstream target of the SCF-c-Kit axis and a definitive modulator of the BM-EPC niche.

### 3.2. Mobilization of EPCs into Injured Tissues

Progenitor cells in a niche are dramatically mobilized to an impaired-tissue region in response to various cytokines. In 1999, Takahashi et al. first studied ischemia- and cytokine-induced EPC mobilization in relation to neovascularization [85]. They generated a mouse model and rabbit model of hindlimb ischemia to test the effect of granulocyte macrophage-colony stimulating factor (GM-CSF) [86] on neovascularization of ischemic tissue. In this model, GM-CSF promotes neovascularization in ischemic tissue, and fluorescent photomicrographs show increased neovascularization in avascular area. Besides GM-CSF, many other cytokines with a specific signaling pathway have been implicated in neovascularization. Accumulating evidence has revealed the importance of various factors including SDF-1, VEGF, placenta-derived growth factor (PIGF), platelet-derived growth factor (PDGF), GM-CSF, and IL-6 [87] in the process of EPC mobilization.

One of the well-known angiogenic cytokines is VEGF [88]. HIF is upregulated under hypoxic conditions [89]. In damaged endothelial cells (ECs) or cancer cells, HIF binds to the promoter region of* VEGF* [90]. EPCs express VEGFR-1 and VEGFR-2 (KDR or flt-1) on the surface of their plasma membrane. Overproduction of VEGF at ischemic sites and in a BM niche leads to EPC mobilization. The VEGFR-VEGF complex activates the phosphatidylinositol 3-kinase- (PI3K-) AKT pathway [91] and thereby is linked to activation of MMP-9, cathepsin, and plasminogen activators, which consequently digest the ECM to promote EPC migration, resulting in enhanced neovascularization [92].

Similarly, stromal-cell-derived factor 1 (SDF-1), also known as CXC motif chemokine 12, is upregulated in ischemic diseases such as myocardial infarction and stroke [93, 94]. Similar to VEGF, stromal SDF-1 is directly targeted and strongly induced by HIF-1*α* because the promoter region of SDF-1 contains a HIF-1*α* binding site. Chemokine receptor CXC motif chemokine receptor 4 (CXCR4) is highly expressed in EPCs [95]. Accumulating evidence reveals that the SDF-1-CXCR4 complex plays a crucial role in EPCs' homing to an ischemic site [96, 97]. SDF-1*α* promotes migration of ECs or EPCs by activating JNK3. eNOS, activated via SDF-1*α*/Akt, produces nitric oxide (NO) and nitrosylates MAPK phosphatase 7 (MKP7), suggesting that eNOS and JNK3 contribute to SDF-1*α*-dependent migration of ECs or EPCs [98]. On the other hand, a CXCR4 knock-out is lethal and exhibit impaired kidney vasculogenesis [99], indicating that SDF-1-CXCR4 axis may play a critical role in EPC mobilization for neovascularization.

### 3.3. Incorporation of EPCs into Damaged Tissues

Incorporation of EPCs into ischemic tissue requires a multiple process [100]. Damaged vascular tissue secretes chemotaxis. According to chemotaxis gradient, EPCs are homing to ischemic region. After that, invasion process is activated. EPCs migrate into vascular tissue, release growth factor, and differentiate into blood vessel cell. To maintain strong structure of blood vessel, adhesion molecules are expressed and connect to neighbor cells.

Homed EPCs are preparing invasion process. Homed EPC and EC, preexisting in vascular tissue, express their surface adhesion molecule. Adhesion molecules expressed on ECs including E-selectin, L-selectin, and P-selectin perform a crucial function in their attachment to ischemic tissues. Both P-selectin glycoprotein ligand-1 (PSGL-1) and sialylated carbohydrate molecules bind to selectin family proteins [101]. Oh et al. demonstrated a pivotal role of E-selectin in EPC-mediated neovascularization [102]. An* in vitro* adhesion assay revealed that blocking of E-selectin using specific antibodies significantly decreases the adhesion ability of EPCs in a gelatin-coated plate. In an ischemic-hindlimb model based on genetically E-selectin-deficient mice, those researchers showed that attachment of homed EPCs to an ischemic limb is severely impaired in E-selectin knock-out mice, whereas injected E-selectin significantly rescued the new vessel formation ability in an ischemic hindlimb of E-selectin knock-out mice. In addition to previous described adhesion molecules, vascular cell adhesion protein-1 (VCAM-1), intercellular adhesion molecule-1 (ICAM-1), and very late antigen-4 (VLA-4 or integrin *α*4*β*1) integrin/lymphocyte function-associated antigen 1 (LFA-1) play important role in this process. VCAM-1 and/or ICAM-1 tightly bind to integrin *α*4*β*1 and LFA-1. This complex facilitates penetration of EPCs into EC-EC connections and transendothelial migration [103, 104]. Upregulated chemotactic signaling molecules stimulate EPCs to secrete MMP-9 or cathepsin L. EPC-EC binding molecules, including integrins, ECM molecules, and ligand-receptor complexes such as SDF-1/CXCR4, are cleaved by MMP-9/cathepsin L (CathL) [105]. Urbich et al. examined CathL expression levels in HUVECs, EPCs, and CD14^+^ cells and showed significantly increased levels of CathL in EPCs [106]. Furthermore, genetically* CathL*-deficient mice (*CathL* ^−/−^) show impaired neovascularization, suggesting that the MMP-9-cathepsin L axis may be strongly involved in recovery of damaged tissues by promoting the EC-ECM interaction.

As a result, newly incorporated EPCs are getting mature and differentiate into EC. Integrins, such as *β*1 and *α*_*γ*_ *β*5, contribute to strong connection between new and old cell [107, 108].

## 4. Cytoprotective Mediators in EPC Biology for Better Cellular Longevity

Most of transplanted EPCs seldom survive under the harsh conditions of ischemia, inflammation, oxidative stress, and mechanical stress [109]. To prevent cell loss and improve the survival rate of EPCs at ischemic sites, tremendous research efforts have eventually uncovered several pivotal cytoprotective mediators relevant to EPC survival in an ischemic environment.

Akt, a serine-threonine protein kinase, is a major regulator of cell growth, differentiation, and cell survival and death. In response to VEGF, SDF-1, angiotensin 1, or reactive oxygen species (ROS), the PH domain of Akt binds to PI3K. Thr308 and Ser473 are sequentially phosphorylated by 3-phosphoinositide-dependent protein kinase 1 (PDK1) and PDK2 and consequently activate the downstream effectors [110]. Hur et al. demonstrated a cytoprotective effect of Akt during EPC-mediated neovascularization [111]. They isolated EPCs from 1,1′-dioctadecyl-3,3,3′,3′-tetramethylindocarbocyanine-labeled mice. When systemically administered, these EPCs can show each stage of homing to an ischemic limb. In ischemic muscle, ischemia induces VEGF expression, SDF-1 activates Akt signaling in ECs and consequently upregulates Akt-enhanced ICAM-1 expression on ECs. Due to the increased expression level of ICAM-1, EPCs can easily intermingle with ECs, suggesting that activated Akt may be sufficient for induction of EPC homing without VEGF or SDF-1. They further demonstrated that Akt gene-transferred ischemic tissue shows enhanced homing of EPCs, vessel formation, blood flow recovery, and ischemic tissue indication, providing that specific regulation of Akt in EPC biology may be a novel therapeutic target in ischemic organs.

Reactive oxygen species- (ROS-) related signals are key cytoprotective mediators that improve cell survival of transplanted EPCs in a harsh ischemic microenvironment. Smooth muscle cells (SMCs) and ECs from injured tissue robustly produce ROS. These highly reactive chemicals then approach the artery wall and react with low-density lipoprotein (LDL). Uncontrolled ROS levels in a blood vessel cause atherosclerosis [112]. Zhang et al. demonstrated the cytoprotective properties of an ROS scavenger in EPC biology using atherosclerosis models [113]. Treatment with oxidized LDL (Ox-LDL) reduces the numbers of circulating MNCs and EPCs, and this effect is attenuated by probucol, an ROS scavenger. Electron paramagnetic resonance data indicate that ROS generated by ox-LDL are eliminated by probucol. In murine BM analysis, probucol-treated hyperlipidemic mice show an elevated number of EPCs. Probucol downregulates ox-LDL and C-reactive protein (CRP) and upregulates superoxide dismutase (SOD), suggesting that specific modulation of ROS by probucol can restore EPC-mediated neovascularization.

Thymosins, a family of small proteins isolated from the calf thymus, have been shown to be involved in pathological conditions, such as wound healing, angiogenesis, and myocardial infarction [114, 115]. Qiu et al. have studied the role of thymosin *β*4 in EPC biology [116]. As in other studies, thymosin *β*4-treated EPCs showed an enhanced migration ability. Thymosin *β*4 stimulates Akt/eNOS phosphorylation and upregulates eNOS signaling in preconditioned EPCs; thymosin *β*4 can also increase the survival ratio of transplanted EPCs via NO-mediated vascular tissue relaxation and augmented blood flow.

## 5. Drug-Based Functional Improvement of Cytoprotective Mediators in EPCs

Statins, cytoprotective mediators well known as 3-hydroxy-3-methyl-glutaryl- (HMG-) CoA reductase inhibitors, are generally used for regulation of cholesterol levels in blood. Because of this lipid-lowering effect, statins are clinically used by patients with myocardial infarction [117], heart failure [118], vasospasms [119], diabetes [120], and hypertension [121]. The study on statin-treated EPCs by Vasa et al. revealed that a statin can improve EPC functional activities [122]. Although patients with CAD show a reduced number of CD34/KDR-positive cells, administration of atorvastatin to these patients leads to an increase in the CD34^+^-KDR^+^ cell population, while there is no significant change in CD34^+^ or CD133^+^ cells. Migratory capacity of EPCs isolated from atorvastatin-treated patients with CVD is significantly augmented, as compared with EPCs from untreated patients. Those researchers also evaluated the serum cholesterol and cytokine levels, which are related to modulation of EPC mobilization or angiogenesis* in vivo.* Statin treatment did not alter serum levels of GM-CSF, TNF-*α*, and VEGF, supporting the hypothesis that statin treatment stimulates the differentiation of endothelial precursor cells into EPCs, suggesting that statins directly enhance the EPC migration ability in patients with CAD.

Hydrogen sulfide (H_2_S) was recently recognized as an important cytoprotective mediator in the cardiovascular system. This colorless and water-soluble gas is produced by catalytic reactions of L-cysteine. Downregulation of H_2_S influences inflammation, circulatory shock, and reperfusion injury in various animal models [123]. For instance, db/db mice as a model of diabetes mellitus were used in studied on H_2_S-mediated diabetic-wound healing [124]. H_2_S significantly stimulates the angiogenic function of EPCs via upregulation of angiopoietin-1 and the VEGF signaling pathway, as well as EPC-mediated wound healing.

eNOS, induced by phosphorylated Akt, generates NO. Released NO activates MMP-9 [125]. This enzyme cleaves mKitL and releases sKitL. Secreted c-Kit (sKitL) promotes cell survival and mobilization and proliferation of EPCs. NO is an essential cytoprotective mediator for cardiovascular homeostasis. This colorless gas inhibits contraction of vascular smooth muscle, platelet aggregation, and leukocyte adhesion to the endothelium. NO also inhibits EPC apoptosis but enhances cell proliferation and MMP9-mediated EPC mobilization [125]. Dysfunction of NO pathways in human is closely related to CVDs including atherosclerosis and hypertension and to diabetes. NO-derived neuronal NOS is well known as a mediator of vascular relaxation in the penis [126]. Erection of the penis is sustained by PI3K/Akt-dependent phosphorylation and activation of eNOS, an NO-producing enzyme [127].

## 6. Promising EPC-Based Therapeutic Strategies against CVDs

Because transplanted EPCs are facing the harsh environment of an injured tissue, including low oxygen concentration, ROS, an inflammatory attack, and nutrient insufficiency, clinical trials of transplanted EPC have yielded unsatisfactory outcomes [128]. In this section, we describe the recently proposed promising strategies for EPC-based cell therapy for severe ischemic CVDs.

### 6.1. Preconditioning of EPCs with Natural Compounds

Development of cell-priming protocols via preconditioning of EPCs may be promising therapeutic strategies for treatment of CVDs. Multiple research groups have attempted to develop novel therapeutic protocols on the basis of findings about cytoprotective mediators, pharmacologically qualified drugs [129], growth factors [130], natural compounds [131], and signal-related triggers for primed cells [132].

Fucoidan, a marine sulfated polysaccharide, is mainly found in extracts from brown algae and brown seaweed [133]. Many researchers have studied the therapeutic effects of fucoidan on osteoarthritis [134], cancer [135], diabetes [124], obesity [136], and kidney [137] and liver disease [138]. To overcome the replicative cellular senescence of isolated EPCs, Lee et al. tested the ability of fucoidan to rescue EPCs from senescence* in vitro* and its vascular repair capacity* in vivo* [139]. EPCs briefly preconditioned with fucoidan caused a significant increase in the number of CD34^+^ EPCs, CXCR4^+^ EPCs, C-kit^+^ EPCs, and VEGFR2^+^ EPCs. Notably, cellular senescence of EPCs is significantly attenuated by regulation of FAK-, Akt-, and ERK-related signaling cascades by fucoidan-treated EPCs. In hindlimb ischemia models, a transplant of fucoidan-treated EPCs results in improved limb salvage with enhanced* in vivo* cell proliferation and cell survival in ischemic tissue.

Tripterine (celastrol) is extracted from the medicinal plant* Tripterygium wilfordii*. This quinone methide is used for treatment of rheumatoid arthritis in traditional Chinese medicine [140]. According to recent studies, tripterine has anticancer [141], antioxidant [142], and anti-inflammatory activities [143]. Lu et al. recently reported the efficacy of tripterine-treated EPCs against atherosclerosis [144]. In an* in vitro* culture assay, tripterine-priming EPCs showed enhanced adhesion, migration, proliferation, and tube formation, as well as a reduced apoptotic ratio during incubation with ox-LDL. By means of short-term priming of those cells with tripterine, the level of integrin-linked kinase (ILK) that is downregulated by ox-LDL is restored, whereas transfection with a siILK and dominant-negative ILK-expressing vector inhibits phosphorylation of ILK's downstream gene protein kinase B (PKB)/Akt and glycogen synthase kinase 3*β* (GSK-3*β*). When injected into atherosclerotic mice, tripterine-primed EPCs decrease aortic lesions and plaque deposition according to hematoxylin-eosin staining of aortic roots, suggesting that tripterine enhances EPC activity against atherosclerosis by means of the ILK signaling pathway.

Accumulating empirical evidence suggests that ovarian hormones such as estrogen and progesterone regulate angiogenesis [145, 146]. Estrogens in women exist in three major forms: estrone (E1), estradiol (E2), and estriol (E3). The predominant form of estrogen during reproductive years is estradiol [147].* In vitro* experiments revealed that estradiol induces EC proliferation [148] and migration [149] and reduces senescence [150]. In this regard, effect of resveratrol is investigated in EPC biology [151, 152]. This polyphenolic phytoalexin, extracted from grapes, has been reported to act as an estrogen receptor agonist [153]. In addition, it also has been reported to enhance expression and activity of eNOS in ECs [154]. When incubated with resveratrol, resveratrol-treated EPCs show increased number of EPCs and promoted EPC proliferation, adhesion, and migration, as well as increased VEGF production and induced vasculogenesis, suggesting that preconditioning of EPCs by resveratrol might be a promising therapeutic strategies for treatment of CVDs. Recently, Lee et al. clearly reported that genistein, an isoflavone derived from soybeans, has affinity for estrogen receptors and strongly improved the engraftment of transplanted EPCs in an acute myocardial ischemia models [155]. When pretreated with genistein, primed EPCs represent enhanced EPC migration and proliferation via increase of ILK, *α*-parvin, and F-actin and phosphorylation of ERK 1/2 signaling, suggesting that pretreatment of EPCs with genistein prior to transplantation can improve the regenerative potential in ischemic tissues, providing a novel strategy in adult stem cell therapy for ischemic diseases.

### 6.2. Genetic Modification of EPCs with Cytoprotective Modulators

To enhance EPC bioactivities, major efforts have been devoted to genetic modification of transplanted cells as a strategy against CVDs. In this subsection, we introduce recently reported cytoprotective mediators, angiotensin-converting-enzyme 2 (ACE2), insulin-like growth factor 1 (IGF-1), and integrin-linked kinase (ILK).

In the renin-angiotensin system, ACE2 converts angiotensin 1 into angiotensins 1–9 and angiotensin 2 into angiotensins 1–7 [156, 157]. With the increasing recognition of their importance in the renin-angiotensin system, ACE2-targeting therapeutic studies are still in progress [158]. The role of ACE2 in EPC function was studied by Chen et al. [159]. They isolated BM-derived EPCs from R^+^A^+^ and R^−^A^−^ mice and then infected these cells with a lentiviral system expressing the* ACE2* gene. Overexpression of ACE2 in EPCs upregulates eNOS but downregulates Nox2 and Nox4. Similarly, NO production by* ACE2*-transduced EPCs is greater than that of control EPCs. An* in vitro* functional assay and an* in vivo* study on a middle cerebral artery occlusion- (MCAO-) induced stoke model further showed that ACE2 enhances EPC function via eNOS/NO and Nox/ROS signaling pathways.

A hormone structurally analogous to insulin, IGF-1, performs an important function in embryonic and postnatal development. Various processes in EPC biology such as cell proliferation, migration, and incorporation are influenced by IGF-1 [160]. Sen et al. genetically modified EPCs with* IGF-1* gene-carrying AAV systems and transplanted EPCs into a rat model of myocardial infraction [161]. Compared to LacZ-EPCs,* IGF-1*-transduced EPCs yield increased cardiomyocyte proliferation and a greater capillary number in the peri-infarct area with a reduced cardiac apoptosis rate, indicating that genetic modification of EPCs by the* IGF-1-*AAV delivery system may be an effective tool for EPC-based cell therapy.

Several researchers have targeted integrin-linked kinase (ILK) for genetic modification of EPCs as a strategy against CVDs, because integrins are located in the cell membrane and regulate cell migration, survival, proliferation, and differentiation. ILK has also been reported to serve as a key regulator of actin rearrangement, cell polarization, spreading on a substrate, migration, proliferation, and survival [162]. Wang et al. generated* ILK* gene-transduced EPCs from subjects with preeclampsia and studied the possible effects of the modified EPCs against CVDs.* ILK-*overexpressing EPCs showed enhanced bioactivities including cell proliferation, migration, and* in vivo* EPC-mediated neovascularization, revealing a good potential for an EPC-based gene therapy in patients with preeclampsia [163].

### 6.3. Strategies for a Combinatorial EPC Cell Therapy

Development of a combinatorial cell therapy using multiple stem cells might be a promising treatment strategy, because various adult stem cells have unique cell lineage determination, offering synergistic effects on cell-based-neovascularization against CVDs.

To develop cell hybrid therapeutic strategies, Lee et al. studied the functional properties of two types of EPCs [164]: CD34^−^/CD34^+^ cell-derived ECFCs (hybrid-dECFCs) and CD34^+^ cell-derived ECFCs (stem-dECFC) [44]. Hybrid-dECFCs showed remarkably enhanced bioactivities such as greater proliferative properties but delayed senescence. In an* in vivo* mouse model of hindlimb ischemia, hybrid-dECFCs showed significantly improved blood perfusion, capillary density, and improved cell survival and proliferation. Particularly, hybrid-dECFCs migration is significantly enhanced via an augmented phosphorylation cascade of focal adhesion kinase (FAK) [165] and Src [155] and consequently results in enhanced incorporation capacity of hybrid-dECFCs, suggesting that the CD34^−^ accessory cells may serve as niche-supporting cells during formation of CD34^+^ cell-derived EPCs.

Zigdon-Giladi et al. recently reported the effects of EPCs in combination with MSCs on bone regeneration [166]. Osteogenically transformed MSCs and EPCs were seeded into gold domes and mixed with a *β*-tricalcium phosphate (*β*TCP) scaffold [167]. Rats with the transplant of the EPC/MSC hybrid cells show increased blood vessel density, vertical bone height, and bone area in the regenerated tissue. Although the groups 4 weeks after transplant had similar characteristics for control and EPC/MSC treatment, the above parameters were doubled in the EPC/MSC hybrid transplant group at 12 weeks after transplant, suggesting that the EPC/MSC hybrid transplant increases blood vessel formation at early stages and enhances bone regeneration at later stages.

Kang et al. attempted to enhance islet engraftment by cotransplanting EPCs with islets [25]. Porcine islets and human cord blood-derived EPCs were cotransplanted into diabetic nude mice [168]. At 11 days after transplant, the islet/EPC group reached euglycemia [169], in contrast to the islet-only group. Insulin levels were found to be higher in the islet/EPC group than in the islet-only group, pointing to rapid revascularization with a VEGF-A-dependent enhanced perfusion ratio. Cell proliferation of *β*-cells is also increased by hepatocyte growth factor, indicating that an islet/EPC transplant may be an efficient therapeutic strategy against diabetes [170].

### 6.4. EPC-Mediated Secretome and Paracrine Effects on Cardiovascular Regeneration

EPCs contribute to cardiovascular regeneration indirectly via secreting tremendous secretomes, including microvesicles (MVs), cytokines, and chemokines, including VEGF, basic FGF, and IGF [171]. These secretomes or cytokines affect itself (autocrine) or other cells (paracrine) [172].

Previously, it has been reported that EPC-conditioned media have cardioprotective role in MI [173]. In addition, soluble factors from EPCs enhance cell function and promote cardiovascular regeneration [174]. Recent study reveals that proteinaceous and nonproteinaceous factors are involved in EPC-mediated cytoprotective role on ischemic insult in brain [175].

In cell-to-cell communication, diverse types of cells secrete microvesicles (MVs) in response to different stimuli from environmental condition [176]. Interestingly, pivotal proteins and RNAs (mRNA, miRNA) are capable of transporting to target cells via MVs. To investigate the role of EPC-derived MVs on cardiac hypertrophy and apoptosis, Gu et al. evaluated Ang II-induced cardiomyocyte (CM) hypertrophy and apoptosis in condition of with or without EPC-MVs [177]. Similar to previous report from EPC-MVs effects on angiogenesis [178], EPC-MVs strongly promote vascularization via effective incorporation into CM, resulting in decreased hypertrophy and apoptosis rate. Importantly, ROS overproduction induced by Ang II was significantly decreased in MVs-CM via regulation PI3K/Akt/eNOS pathway [179].

### 6.5. Tissue Engineering Strategies to Facilitate EPC-Mediated Neovascularization

To improve EPC function, continuous efforts of investigators recently provided novel therapeutic strategies involving biocompatible biomaterials such as metallic compounds, polymers, and composite materials against CVDs [180].

To prevent the complications such as local thrombus formation and restenosis, Shirota et al. fabricated two types of EPC-seeded intravascular stent devices [181]. A photocured gelatin-coated metallic stent and a photocured gelatin-coated microporous thin segmented polyurethane (SPU) film-covered stent were used in this experiment with a confluent monolayer of seeded EPC [182, 183]. Tubular hybrid vascular medial tissue, composed of vascular smooth muscle cells and collagen, was filled with expanded EPCs. Migrating EPCs from the stent struts proliferated and endothelialized the luminal surface of the hybrid vascular medial tissue [184]. On-stent delivery EPCs may be a novel therapeutic approach to reconstitution of the atherosclerotic arterial wall with various benefits, prevention of thrombosis and restenosis [185], and promotion of rapid formation of normal tissue architecture.

Peters et al. reported efficacy of hydrogel scaffolds in terms of EPC-mediated neovascularization [186]. This biomaterial consists of a cell-adhesive and proteolytically degradable polyethylene glycol hydrogel [187]. EPCs are cocultured with angiogenic mural cells (SMCs) in encapsulated hydrogel scaffolds. Polymeric precursors were mixed with the cells, and then a mild photo-cross-linking procedure was performed [188]. Without additional cytokines, EPCs formed a 3D microvessel network. After 2 weeks of coculture, those investigators observed 3D-EPC microvessels, suggesting that initiating custom hydrogels may support formation of diverse microvessels.

## 7. Future Directions

**Figure 2:**
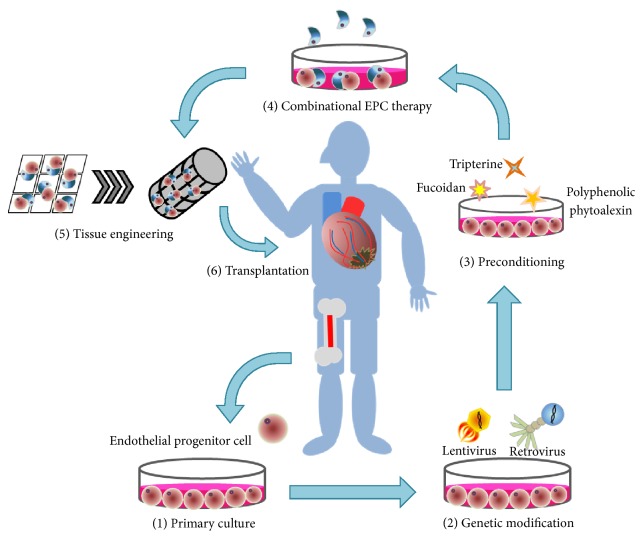
Promising therapeutic strategies for EPC therapy. Multistep combinational therapeutic strategies of EPC therapy against CVDs include the following: (1) patient-derived EPCs are isolated from healthy tissue; ((2), (3), (4)) improvement in EPC bioactivities via genetic modifications or pretreatment with natural compound or cocultured with other stem cells, such as MSCs and CPCs; and ((5), (6)) tissue engineering techniques using biomaterials protect cells from the harsh conditions of ischemic regions.

Given the increasing life expectancy, prevention and management of CVDs in humans are necessary. In the human body, the blood circulation system is omnipresent and connects different tissues. To recover injured vessels, the understanding of pathophysiological niche components and their supporting cells including stem/progenitor cells is crucial. In terms of EPC-based cell therapy, it may useful to screen the promising cytoprotective mediators in experiments on EPC biology. Specific target molecules are prestimulated by natural products, with a genetic modification, or tissue engineering technologies to promote cell-ECM interactions for better EPC bioactivities, before a transplant into an ischemic tissue. Due to their complexity, EPC-based regeneration strategies should be integrated; this way, they will still have a tremendous regenerative potential in CVDs. Further experiments on EPC-based therapeutic strategies can open up more promising opportunities, where a combination of multiple strategies may improve EPC bioactivities including cellular proliferation, survival, and* in vivo* differentiation (Figure 2).
